# Sex Offender Recidivism: Some Lessons Learned From Over 70 Years of Research

**DOI:** 10.1177/07340168231157385

**Published:** 2023-02-21

**Authors:** Patrick Lussier, Stéphanie Chouinard Thivierge, Julien Fréchette, Jean Proulx

**Affiliations:** 1 School of Social Work and Criminology, 4440 Université Laval, Quebec, Canada; 2 Centre International de Criminologie Comparée, Quebec, Canada; 3 School of Criminology, 5622 Université de Montréal, Montreal, Quebec, Canada

**Keywords:** sex crimes, corrections, crime policy, evaluation research, quantitative methods, sexual recidivism

## Abstract

Sex offender recidivism (SOR) has been the subject of research for over 70 years. Myths, misconceptions, and erroneous conclusions about SOR, however, remain widespread, impeding the development of evidence-based policies aimed at preventing sexual offenses. To address the rich but uneven literature, a comprehensive review was conducted making it possible to provide a contextualized overview of scientific knowledge against the backdrop of methodological issues, challenges, and shortcomings. Over the years, researchers have been asked to provide a simple answer to a seemingly simple question: what are the recidivism rates for sexual offending? In response, the field has produced a wide range of findings making it difficult to draw firm conclusions, leaving room for interpretation and personal biases. The variations in recidivism rates are attributable to offender and methodological characteristics, both of which are embedded in a particular sociolegal context. As a result, the base rate of SOR is more effectively considered in terms of a series of questions that should include the type of recidivism, with whom, over what period, and in what context. Issues and debates that have marked the field and fueled its growth are highlighted. Research innovations and important areas of research are also discussed.

Every generation seems to define particular sets of behaviors as social problems (e.g., Blumer, 1971) and identify the groups involved as folk devils (Cohen, 1972). In North America, since the 1940s, these behaviors and their perpetrators have included sexual offenses and sexual offenders, defined in ways that led to three waves of sociolegal responses: the clinical-medical model, the sociolegal-feminist model, and the community risk-protection model (e.g., Lieb et al., 1998; Petrunik, 2002). We have now entered a fourth wave, sparked in part by the *#MeToo* movement and its focus on certain sexual offenses (e.g., sexual misconduct, sexual harassment, sexual trafficking) by individuals in positions of power (e.g., Lussier et al., 2021b). Sexual offending phenomena include a broad spectrum of behaviors but at any given time only some are defined as deserving of government attention and intervention. In the 1990s, following a series of rare but brutal events, the existence of “the sexual recidivist” began to be seen as an urgent social problem in need of immediate attention and intervention by policymakers (e.g., Simon, 1998) and laws tailored toward prevention of sexual offending by individuals previously convicted of a sex crime became part of the agenda of many criminal justice agencies. This attention led to legal changes and policy developments in the criminal justice system that affected individuals labeled as “sex offenders.” Research in this area has increased over the years, and researchers are now closer to answering questions raised some 30 years ago by policymakers and stakeholders, prosecutors and lawyers, psychologists and psychiatrists, victims, victims’ rights advocates, and journalists. However, despite current social movements and media attention to certain sexual offenses, the question of how to define and deal with “sexual recidivists” is still at issue.

In North America, while the 1990s was a pivotal point for the development of policies and measures aimed at managing the risk of recidivism by sex offenders, the issue had caught researchers’ attention several decades earlier (e.g., Tappan, 1950). Research in this area has been uneven, limited in scope, and plagued by methodological issues and limitations, but its history, evolution, and current status merit more attention. First, there are still misconceptions, myths, and erroneous beliefs about sex offender recidivism that are not limited to the general public but affect criminal justice practitioners, professionals, and policymakers. These myths and misconceptions contribute to the idea that sex offenders constitute a distinct group whose members are more unpredictable, more irrational, and less likely to change than other offenders (e.g., Weekes et al., 1995). Such individuals are often thought of as on a path of increasingly serious and violent sexual offenses, culminating in sexual homicide. Second, despite the existence of a large body of research, its uneven quality has led to study findings that are unclear and ambiguous. For instance, several researchers over the years have stressed that sex offender recidivism (SOR) rates are overestimated by the general public and policymakers. Third, while there have been other reviews of research on sex offender recidivism (e.g., Furby et al., 1989), these have generally been oriented around two main questions: (a) What is the base rate of sexual recidivism among individuals convicted of a sex crime? (b) Are treatments for sex offenders capable of reducing these rates? We believe that this approach is not only too limited and restrictive but is based on assumptions about the risk of sexual recidivism that need to be examined. Therefore, given the backdrop of myths and misconceptions, seemingly contradictory research findings, and a relatively narrow approach to a complex issue, we feel that a narrative review of the scientific literature on this subject can make a significant contribution to the field.

## Purpose of the Study

The criminal justice system expects criminal justice professionals, expert witnesses, and researchers to have at least some understanding of the danger posed by an individual convicted of sexual offenses. However, clinical assessment of risk is often based on criteria that stem not from scientific consensus but from legal considerations (e.g., Donaldson & Wollert, 2008). Conducting a risk assessment and making some form of risk prediction have, over the years, become almost routine for courts, correctional institutions, parole/review boards, and in civil commitment situations (e.g., Frechette & Lussier, 2021). The stakes of these risk assessments are high, for both the individual and society (e.g., Janus, 2000). When a convicted offender sexually reoffends, the legitimacy of the criminal justice system is scrutinized, questioned, and criticized (see Sutherland, 1950). The pressure to make such assessments exist irrespective of whether it is based on realistic expectations about what professionals can and cannot do and is sometimes greeted with doubt, skepticism, and criticism, especially when professional risk assessments seem insufficiently decisive or precise, or prove to be inaccurate. Risk assessment is heavily dependent on the quality of the scientific knowledge and instruments available to professionals conducting these assessments as well as the ability to use research on these issues with professional judgment and discernment. A central part of the assessment of risk rests on a key, although imperfect, measure: sexual recidivism.

Sexual recidivism has been investigated for years by researchers from various backgrounds, creating an impressive amount of scientific knowledge. The scientific literature on SOR now spans over 7 decades and, in the process, numerous issues, challenges, and debates have characterized this field of research. While several policy analyses, reviews, and meta-analyses (e.g., Hanson & Bussière, 1998; Soothill, 2010; Lussier et al., 2022; Lussier et al., 2023a) have been conducted on SOR and related issues, we believe that a comprehensive and critical overview on SOR research and its evolution is lacking. The need for a comprehensive overview of SOR research is supported by the fact that this literature is: (a) dense and complex, producing seemingly contradictory findings that may be difficult to translate into criminal justice policies; (b) fragmented across academic disciplines, research interests, and research focus; (c) evolving over time, characterized by a unique and cumulating knowledge base which needs to be properly situated. The purpose of this study, therefore, is to conduct a comprehensive review of the scientific literature on SOR by examining what we know, what we do not know, and should know about SOR. We believe that an understanding of this knowledge requires a thorough examination of SOR research and how it has evolved over time and context.

## Review Search and Selection Process

We agree with Soothill (2010) that an effective review of the scientific literature on SOR requires a legal and sociohistorical context that is beyond the scope of a systematic review. While a systematic review is useful to address a specific research question, a narrative review can provide a wider overview of the research on a given topic. This type of review, however, often involves the loss of detailed information about particular methodology and study findings. Our narrative review is therefore supported by information collected as part of a systematic review (e.g., see Lussier et al., 2022), a combination of review methods that has been shown to be of value in addressing complex research questions (e.g., Contandriopoulos et al., 2010). Using Gold Rush software, a library content comparison tool, the following databases, chosen because they minimized overlaps, were selected to conduct the literature search: Academic search premier (EBSCO), Criminal Justice Abstracts (EBSCO/Proquest), Embase (Elsevier), ERIC (EBSCO), International Bibliography of the Social Sciences (ProQuest), Law Journal Library (HeinOnline), Legal Source (EBSCO), Medline (Ovid), NCJRS (ProQuest), PsycInfo (Ovid), Sociological Asbtract (ProQuest), and Web of Science. Because of the importance of “gray literature” (i.e., unpublished material that is not included in typical computerized retrieval systems), the search was expanded to several additional databases^1^ to minimize the impact of publication bias and its impact on our findings (e.g., Conn et al., 2003)

**Figure 1.**
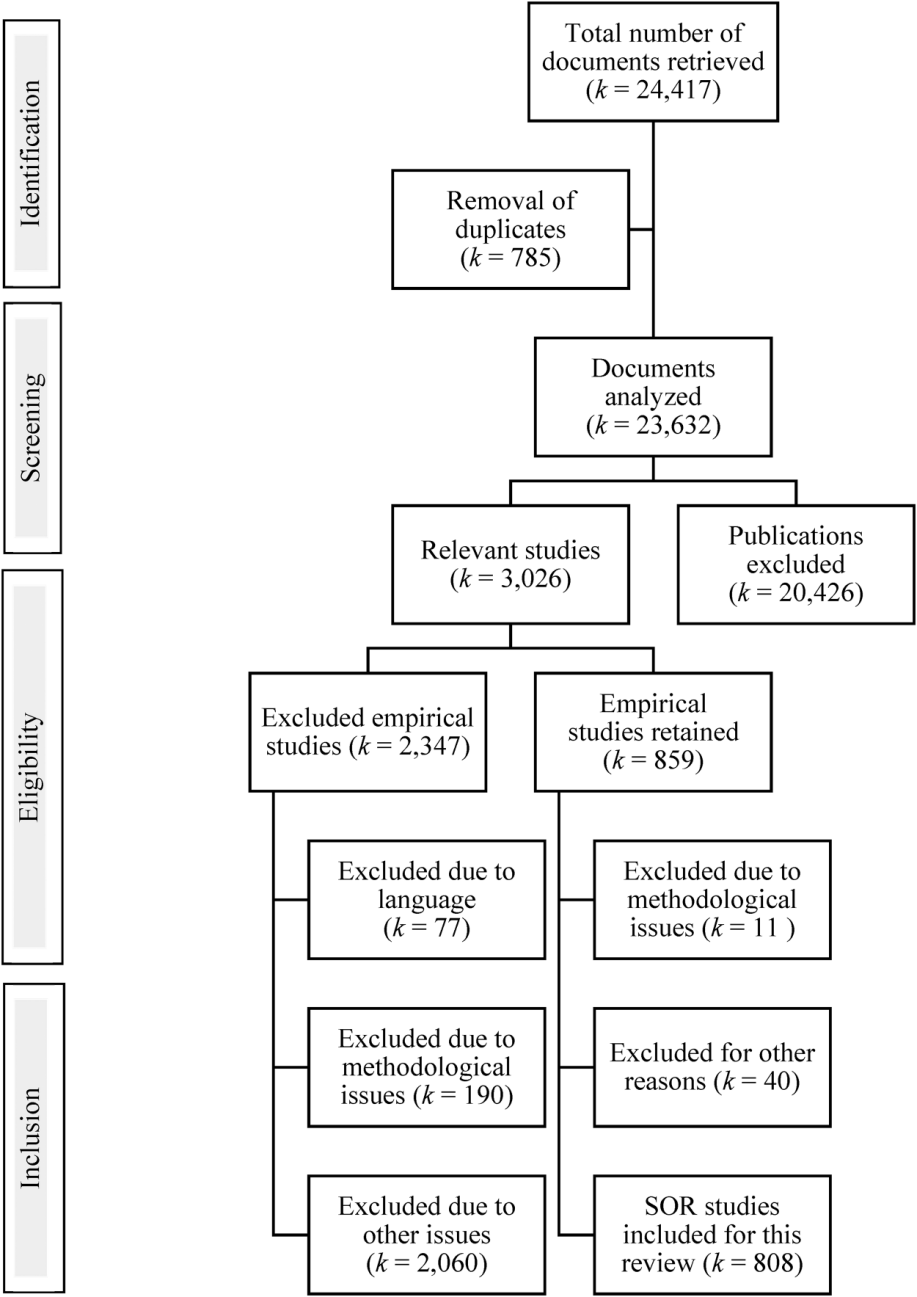
Flowchart for empirical studies on sex offender recidivism.

Using a combination of search terms (see Appendix I), a total of 24,417 references were retrieved. Removal of duplicates reduced the number to 23,632 (see Figure 1). At this stage, research assistants were instructed to identify documents that potentially included an empirical assessment of recidivism rates for a sample of offenders. They were also instructed to screen studies that potentially included a sample of individuals with histories of sexual offending. To do so, research assistants examined the document's title, the abstract, and all keywords to determine if a publication was potentially an empirical study dealing with SOR. In doubt, research assistants were instructed to keep the document for the next stage which involved a complete examination of the document. This stage revealed how varied this scientific literature is (e.g., policy analyses, case studies, descriptions of a risk assessment tool, reviews of the risk factors of sexual recidivism, etc.). At the end of this stage, 20,426 documents were removed. A total of 3,206 studies were examined more fully by the research team to determine whether the publication provided information on collecting, analyzing, and reporting data on recidivism rates for a group of individuals labeled as sex offenders, identifying it as an empirical study rather than, for example, a narrative review or a meta-analysis of the topic. This involved retrieving a copy of the document and examining it thoroughly. This filtering process led to the removal of 2,347 studies. Among those removed were studies written in a language other than English or French, those for which the full report could not be located or accessed, and those that were not based on a prospective longitudinal research design. This left 859 studies that met our criteria for inclusion: the report, written in English or French, had been published after 1949, could be accessed in full, and was an empirical study based on a longitudinal research design that included a sample of sex offenders and provided data on criminal recidivism. After further examination, 51 additional studies were removed from the final sample because, for example, recidivism rates were not reported specifically for a group labeled as sex offenders or a retrospective study design had been used. This process led to 808 empirical studies of SOR being retained for examination.

These 808 empirical studies were read, analyzed, and coded by a team of trained research assistants using a coding sheet. A detailed description of the methodology and the coding of the studies can be found elsewhere (see, Lussier et al., 2022; 2023a; 2023b). An interrater analysis (*k*  =  150) was conducted on each of the variables examined by pairs of coders who were unaware of the research questions and kappa coefficients of reliability are reported throughout. For the purposes of this study, a limited number of factors were analyzed to provide a better sense of the basic characteristics of SOR research over the years. Additional information was also collected, including the year of publication, geographical location of the study, measures and coding used to determine recidivism, nature of the sample and sample size, and the availability of information about the methodology used. This complementary approach provides baseline information about SOR research, focused on five fundamental topics: (a) the context of SOR research and its evolution over the years, (b) information provided by the empirical studies, (c) unresolved issues around SOR, (d) methodological issues, challenges, and limitations that characterize the study of SOR, innovations and scientific advancements in the measurement of SOR.

## Sex Offender Recidivism Research in Perspective

The origin of the term recidivism can be traced back to the Latin word *rĕcĭdīvus*, which literally means to fall back—that is, to fall back into old habits or into a previous condition. While the terminology has changed over time (from, for instance, failure, relapse, violation), the term recidivism is already found in the scientific literature on SOR in the early 1950s. The term “criminal recidivism” combines two related components: (a) the repetition of criminal behavior (i.e., a behavioral component); (b) the persistence of this behavior over some period (i.e., a temporal component). It is generally examined using criminal justice data (e.g., arrest, conviction) for cohorts of individuals who have been arrested or convicted of a crime. “Sexual recidivism” refers to a repeat arrest or conviction for a sexual offense. The measurement of recidivism typically begins once offenders are back in the community and at risk of reoffending. While at first glance this approach seems straightforward, the concept of recidivism and its operationalization for research purposes is not (see Lussier & Cale, 2013). Given this, the evolution and nature of research on SOR need to be contextualized before the empirical and methodological issues are discussed.

Interest in the study of sexual deviance and, by extension, sexual recidivism can be traced back to the early writings of 19th-century European physicians on “sexual perverts” in asylums (see Lussier et al., 2021a). Sexual deviance was loosely defined as repetitive and persistent sexual behaviors that were deemed odd, atypical, and outside the norm for natural instincts (e.g., Kraftt-Ebing, 1886), and such behavior was not seen as providing information about underlying conditions that might have been influential in its occurrence (e.g., Gley, 1884). Indeed, early clinical work suggested that such individuals had physical and mental problems (e.g., psychological degeneration, psychosis) that explained the comorbidity of their actions with problem behaviors that were not sexual in nature (e.g., obsessions, rituals, pyromania, impulsive behaviors). Studies of single cases were used to suggest that sexually deviant behaviors paralleled mental health problems (Magnan, 1885) and could be either sudden and irregular or chronic, repetitive, and increasingly serious over time. These early writings had a significant influence on early clinical practices in North America (e.g., Abrahamsen, 1950; Karpman, 1951; Reinhardt, 1957) and provide the foundation for several assumptions that remain influential in SOR research, such as belief in the specificity of the etiological roots of sexual offending and a proclivity to repeat sexual offending behaviors. SOR research has, however, evolved and it is possible to distinguish between different periods that characterize this evolution. Note that these periods are not as clearly delineated as this review suggests (i.e., certain studies are at the margin of the popular perspective of a period). However, they are useful in understanding the changes in the research on sex offender recidivism over the period considered in the present study.

**Figure 2.**
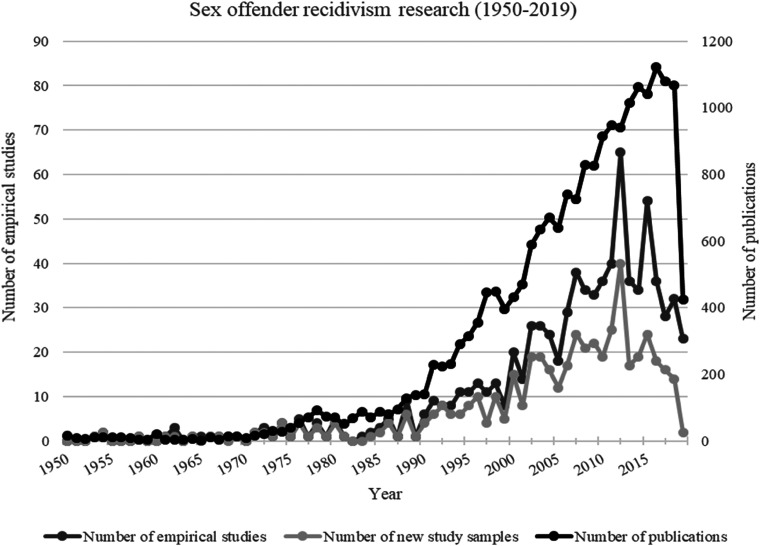
Number of publications and empirical studies on SOR per year (1950–2019).

*Notes.* The leftaxis refers to the number of empirical studies (including the number of new samples used) while the right axis refers to the total number of publications meeting our initial search criteria. The number of publications refers to all instances where our search terms were mentioned in a publication (including gray literature), excluding duplicates. The number of empirical studies refers to those meeting our search criteria (see text). The number of new study samples refers to all new instances where a distinct sample was used by a researcher/team of researchers which included the measurement of recidivism rates (including or not sexual recidivism). The search was done in May 2019, and, therefore, the numbers for 2019 do not reflect the full year (which explains the sudden drop).

Figure 2 shows the yearly distribution of the total publications retrieved (*k*  =  23,632) and the number of empirical studies on SOR (*k*  =  808) from 1950 to 2019, highlighting the slow evolution of research in this area until the early 1970s, when the number of publications increased although the number of empirical studies on SOR remained relatively small. In the 1990s, the number of empirical studies began to steadily increase until the 2010s, peaking in 2012 and followed by a slow decline. It is interesting to note that the rise in the number of publications somewhat mirrors the increase proportion of team-authored relative to solo-authored publications within social sciences (Wuchty et al., 2007). The growth of knowledge in SOR research might have prompted a collaboration between researchers with different research expertise (e.g., clinical assessment, statistics, correctional psychology, criminological theory) in a way that contributed to the growth of the field. Careful consideration of the literature between 1950 and 2019 suggests that there were five key periods indicative of significant changes in SOR research with regard to assumptions, study goals, research methodology, and empirical considerations. Our description of research on sexual recidivism begins with the period 1950–1970, which was characterized by the emergence of laws regarding sexual psychopaths.

### Sexual Recidivism as One of Many Indicators of Sexual Deviance (1950–1970s)

After the Second World War, research on SOR in North American was focused on identifying and delineating the clinical profile and mental health problems of the “sex offender”—that is, an individual charged/convicted of a sex crime (e.g., Abrahamsen, 1950). The underlying assumption at the time was that a prison sentence would not deter such an individual from sexually reoffending and therefore individualized psychological interventions, such as psychotherapy tailored toward mental health problems, were to be preferred (e.g., Guttmacher & Weinhofen, 1952; Hacker & Frym, 1955). These assumptions favored the enactment of “sexual psychopath laws,” which were quickly challenged by criminologists as based on myths and misconceptions about the perpetrators of such offenses (e.g., Sutherland, 1950; Tappan, 1950). Despite this criticism, clinical researchers remained focused on identifying clinical indicators that suggested which mental health problems were responsible for sexually deviant behavior and should be addressed in the rehabilitation process (e.g., Karpman, 1951; Reinhardt, 1957). A number of such indicators were proposed and there was no real consensus about what the key factors were, how to best measure them, or their actual impact on sexual recidivism. Researchers were forced to work blind, given the relative absence of empirical research such as longitudinal studies. What research there was on sexual recidivism focused on descriptions of offenders’ criminal histories using retrospective data (Gebhard et al., 1965) rather than trying to identify risk factors or establishing base rates for sexual recidivism. A filtering process in the criminal justice system and the practice of using samples consisting of individuals on psychiatric wards meant that research participants were usually offenders convicted under the laws aimed at sexual psychopaths and were likely to have an extensive history of sexual offending. The study of sexual offending was therefore limited to observations of sexual recidivists, often those with mental health problems. The resulting picture was that individuals convicted of a sex crime were specialists (i.e., individuals repeatedly and exclusively involved in sex crimes) who had significant mental health problems, particularly those related to sexual deviance (e.g., deviant sexual preferences).

### An Emerging Field of Research in Need of More Objective Measures (1980s)

In the 1980s, the term SOR began making its way into research on sexual offending. By the end of the preceding period, dominant views on psychiatry and medical research on mental health were beginning to be challenged (e.g., Szasz, 1976). Against the backdrop of this social movement, sexual psychopath laws deemed too vague and controversial were abandoned as failed social experiments (Group for the Advancement of Psychiatry, 1977). Such criticism was supported by empirical observations in a series of longitudinal studies of individuals released from psychiatric wards following Supreme Court judgments (e.g., Cocozza & Steadman, 1976). These studies found relatively low rates of recidivism (mainly involving nonviolent offenses) among individuals once deemed too dangerous to be released and sparked a series of legal changes and research innovations in the clinical assessment of risk (e.g., Quinsey et al., 1975; Quinsey & Maguire, 1986). Because the general crime rate was rising to historically high levels in several countries, including sexual offenses (e.g., Ouimet, 1999; Tonry, 2004), governments were pressured to act in a context of much uncertainty about the policy path to take to curb the crime rate (Lussier et al., 2023b). Contrasting interpretations about the causes of the crime rise were made in the United States and Canada leading to very different criminal justice policies (e.g., Tonry & Farrington, 2005).

**Figure 3.**
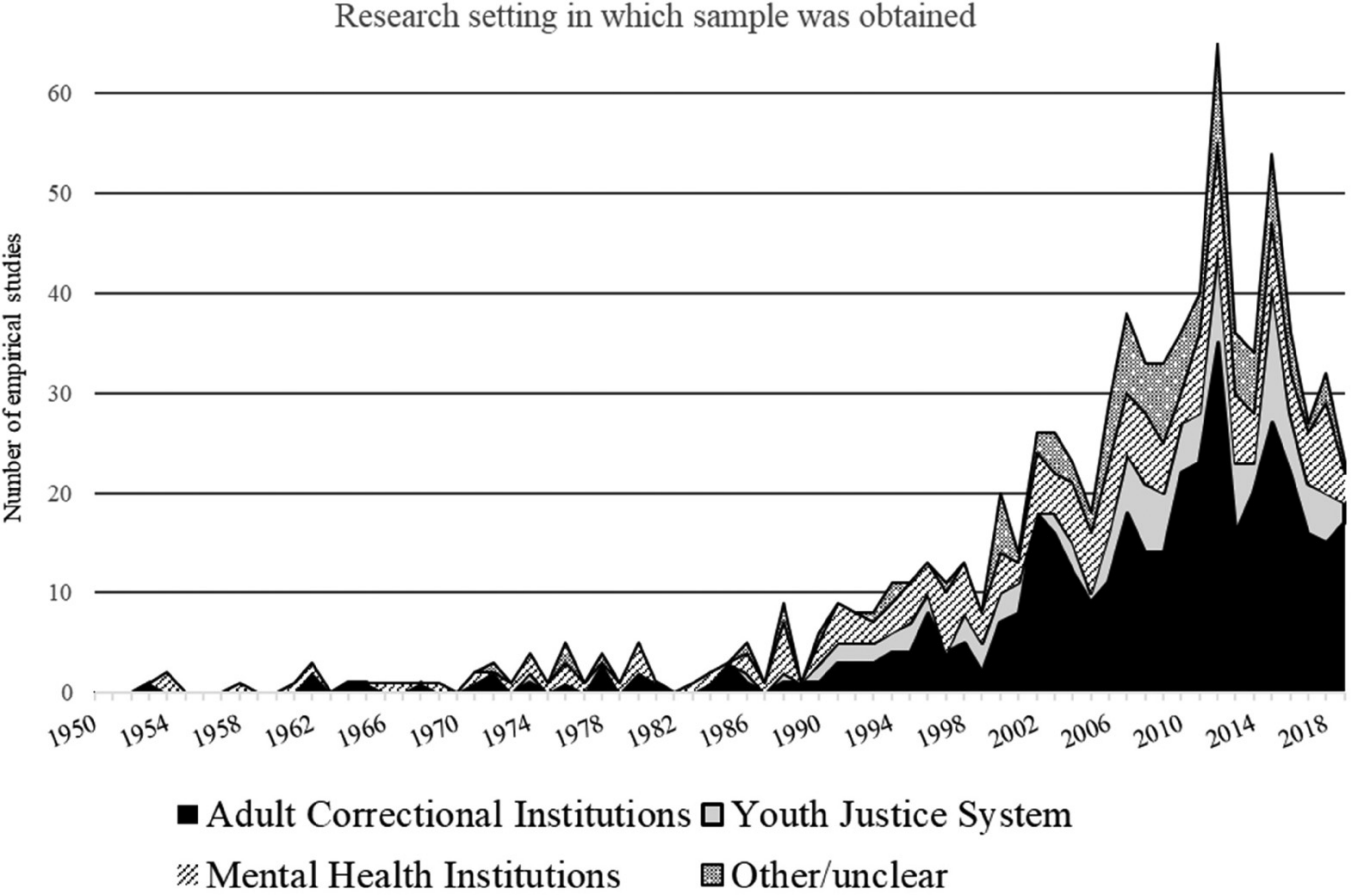
The type of research setting per year of study publication (1950–2019).

*Notes.* These numbers are based on the 808 empirical studies on recidivism, using the criteria discussed in the text. Publications based on the same study data or conducted using the same sample have not been excluded. The search was done in May 2019 and therefore numbers for 2019 do not reflect the entire year. The kappa coefficient was .674 (SE =.051).

Sparked in part by the Martinson's “nothing works” report, the American criminal justice system turned away from individualized sanctions (e.g., see Coffee, 1975) and rehabilitation principles in favor of a deterrent-focused perspective that would lead to a dramatic increase of incarceration rates. This policy shift was supported in part by criminal career research aiming to identify chronic offenders and career criminals. In fact, while criminologists were struggling with how to define, measure, and interpret the concept of recidivism (e.g., Maltz, 1984), others started exploring more fully criminal career parameters (e.g., Blumstein et al., 1986). In Canada, the negative consequences of incarceration were considered as a contributing factor of the general crime rise and various public inquiries recommended that alternative sanctions to incarceration should be more prominently used while reaffirming the importance of treatment/intervention and rehabilitation as a key principle of sentencing (e.g., see Gendreau et al., 1996; Gendreau et al., 1999). A group of Canadian researchers recognized that the rehabilitation principle needed to be anchored in scientific (evidence-based) principles (Andrews et al., 1990a; Andrews et al., 1990b; Andrews et al., 2006). These principles, known as the risk–need–responsivity (RNR) model of service delivery, valued and recognized the importance of risk assessment focusing on static (historical) and dynamic (changeable) risk factors that have been shown to be statistically related to recidivism. That model was adopted in Canada and some U.S. states. Against the backdrop of these significant policy changes, the growing field of sex offender research was looking for ways to establish its legitimacy. While aware of the limitations of the concept of recidivism, Furby et al. (1989) saw this measure as an improvement over other clinical indicators at the time, which they felt lacked both conceptual clarity and validity. In debates over whether treatment was effective (e.g., Marshall & Barbaree, 1988), researchers followed Furby et al.'s recommendations by increasingly focusing on sexual recidivism as a more objective measure of treatment success. Furby et al.'s (1989) recommendations were also aligned with RNR principles in corrections stipulating the importance of identifying changeable risk factors that were predictive of recidivism for treatment/intervention purposes. The notion of risk had not really entered the narrative and sexual recidivism was seen as the most convenient and objective measure of sexual offending. But the convergence of treatment evaluative studies using sexual recidivism as the outcome measure and the emergence of RNR principles in corrections would change that (e.g., see Hanson et al., 2009). While prior research had been conducted primarily using small and biased samples from forensic and mental health settings, SOR research was about to be extended to broader samples involving offenders in prison settings who had been convicted of a sexual offense (Figure 3).

### Sexual Recidivism as an Indicator of Risk (1990s)

The idea of sexual recidivism as a measure of “risk” entered the narrative during the 1990s, part of a shift due to the “new penology” movement in corrections (Feeley & Simon, 1992) and the rise of “preventive detention” in various countries (for a review, see Harrison, 2017). Western societies had undergone significant social changes, leading to a shift in policies associated with SOR from those based on mental health to those based on the notion of risk and risk management (see Beck, 1992). Individuals convicted of a sex crime, especially those returning to the community after completing their sentence, became associated with the idea of risk, leading to the gradual development of a focus on sexual recidivists by researchers in both the field of correctional psychology and criminology (e.g., Hanson et al., 1993). The 1990s also saw the rise of laws aimed at sex offenders and intended to manage risk. While the debate over the efficacy of treatment for sex offenders was still raging (e.g., Quinsey et al., 1993), the idea of rehabilitation was gradually replaced by risk management. Even the treatment models and programs that emerged during this period were closely tied to the notion of risk management (e.g., relapse prevention; Pithers, 1990), and sexual recidivism was increasingly associated with the idea of long-term and static risk that had to be managed by the criminal justice system. This movement toward the idea of risk led to a growing interest in determining the “true” base rate of sexual recidivism and coincides with the acceleration of empirical research on SOR (see Figure 2). Researchers struggling to determine rates of sexual recidivism were increasingly aware of the methodological shortcomings of this measure, but that did not stop them from attempting to establish “base rates” for offender subgroups. This research agenda took two main paths. The first was based on the idea that the type of sexual offending reflected sexual deviance and that sexual recidivism rates could vary accordingly—for example, child molesters/pedophiles, rapists/sexual aggressors of women, exhibitionists, voyeurs—would exhibit different, and determinable, rates of recidivism (e.g., Proulx et al., 2000). The second path, influenced by the writings of Monahan and Steadman (1994) among others, focused on offender characteristics, also known as recidivism predictors, associated with different rates of sexual recidivism (e.g., Proulx et al., 1997; Quinsey et al., 1995) and eventually led to the development of actuarial risk assessment instruments for determining the risk of sexual recidivism.

### Sexual Recidivism: A Measure for All Purposes (2000 − 2010)

During the first decade of 2000, there was a consensus that sexual recidivism, despite its methodological shortcomings, was a necessary and almost inescapable measure. This consensus was accompanied by the belief that researchers had a good idea of what sexual recidivism rates *should* look like (e.g., Hanson et al., 2003), although there had not been any improvements or innovations regarding measurement. The consensus was so widespread that sexual recidivism became a benchmark measure of success. Researchers repeatedly used the same study samples to explore various related issues, using sexual recidivism as an all-purpose measure (see Figure 2). Empirical studies went far beyond providing a description of base rates and associated risk factors, with sexual recidivism used as an objective measure to (a) calibrate and validate risk assessment tools, (b) examine and explore the efficacy of sex offender treatment programs, (c) identify clinical and dynamic risk factors, (d) determine the deterrent impact of newly implemented legal policies targeting individuals convicted of a sex crime (e.g., sex offender registries, public notification, intensive supervision), and (e) as a way to establish the effect of characteristics such as age and sex on levels of sexual offending (e.g., Barbaree et al., 2001). Up to this point, this line of research had focused on the study of adult males, but researchers now extended it to other populations, especially adolescents (e.g., McCann & Lussier, 2008) and adult female offenders (e.g., Cortoni et al., 2010). However, because sexual recidivism was used as a benchmark measure for various research questions and policy issues, there was little research on its validity and few questions about its use. These evaluative studies, therefore, reflect the limitations of the measure of sexual recidivism being used. The failure to consider these limitations had a noticeable effect on the conclusions drawn in these studies (Study limitations are discussed below).

## Challenging the Risk Paradigm (2010s –)

The risk paradigm faced serious criticism for its portrayal of all individuals convicted of sex crimes as having fixed and continuing propensities to commit sexual offenses (e.g., Soothill, 2010), particularly given the relatively low recidivism rates reported in numerous studies. Research in three key areas challenged the assumption of a static and fixed propensity to offend. The first was the issue of offender age—the idea that risk of sexual recidivism is age dependent had been overlooked in previous research (e.g., Barbaree et al., 2003), and work in this area became something of a precursor to the ideas and research that followed (e.g., Lussier & Healey, 2009). Second, the period also saw an increased interest in study samples that were not limited to those in correctional and mental health institutions (e.g., community-based samples) (Figure 3) and longitudinal research using birth cohort data challenged previous estimates of sexual recidivism for youth in correctional institutions (e.g., Zimring et al., 2007). Examining recidivism rates across developmental periods raised clear challenges to the idea of a static propensity to offend for all offenders. Third, these trends led a group of researchers to examine concepts other than recidivism through the study of offending trajectory (Lussier et al., 2010). This work, influenced by the ideas of life-course criminology, was concerned with the dynamic aspect of human lives and how life events and turning points both shape self-identity and provide opportunities that are necessary conditions for significant life changes (e.g., Farmer et al., 2015; Harris, 2014). The life-course perspective is concerned with within-individual changes across the life course and life stages and how this could impact offending and recidivism (Lussier & Davies, 2011; Lussier, 2017). Ideas about the role of age in SOR, put in the context of a life-course perspective, supported increased interest in offending trajectories (e.g., Cale et al., 2016; Francis et al., 2014; Lussier et al., 2010; Lussier & Davies, 2011; Maes et al., 2019; McCuish et al., 2016; Reale et al., 2020; Tewksbury et al., 2012; Thompson et al., 2013) and even influenced researchers to reconsider treatment models based exclusively on risk-based intervention and make room for consideration of protective factors and how to promote offenders’ strengths to encourage and facilitate community reintegration. Concerns over the importance of within-individual-changes across the life course also led researchers involved in long-term risk prediction to reconsider whether a person's level of risk remains stable over time (e.g., whether a high-risk offender always remains at a high risk to reoffend) (e.g., Thornton et al., 2021).

The reliance on sexual recidivism as the gold-standard measure of risk and sexual offending was also challenged, particularly following research on the dynamic aspects of risk, sexual offending, and human lives. The field of correctional psychology stressed the importance of dynamic risk factors responsible for criminal recidivism (e.g., Hanson & Harris, 2000), and developmental and life-course researchers emphasized the dynamic aspects of offending (e.g., Lussier et al., 2021a). Central to the paradigm shift is the idea that the risk of sexual reoffending is more dynamic than previously thought: it can change over time and across developmental stages and can fluctuate according to life circumstances. However, the novel ideas proposed by these two fields are difficult to merge from a practical standpoint. Dynamic risk factors (e.g., self-control, cognitive distortions) can change over a life course, while developmental and life-course research suggests that the nature of risk factors can change across developmental stages and life transitions (e.g., adolescence, emerging adulthood, etc.). The former stresses the importance of specialized treatment and intervention while the latter emphasizes the role and importance of positive life events, key turning points, and the prosocial influence of social networks (e.g., family members; e.g., Kras, 2019; Walker et al., 2020). These ideas were not immediately embraced in all areas, as demonstrated by the introduction of laws dealing with perpetrators of sexual offenses (e.g., sex offender registration, public notification, public sex offender registries, residential restriction laws) based on the perception that the risk of sexual recidivism is high and relatively stable over time. While emerging research suggests that there may be a paradigm shift in criminal justice responses to preventing sexual reoffending, it is important that this shift be built on scientific knowledge about SOR.

## Sex Offender Recidivism: What We Know

The proliferation and evolution of research on SOR saw researchers coming to consensus on a number of issues. As a result, some widely accepted conclusions about SOR are no longer the subject of much debate. While these issues are not conclusively decided, researchers now tend to agree that they should be dealt with based on available scientific evidence.

### There Is No Such Thing as a “True” Base Rate

There is no easy answer to what the risk of sexual recidivism is for an individual convicted of a sex crime or how this risk should be determined. Several researchers have reviewed the literature to attempt to identify statistical trends that would make it possible to characterize the base rate of sexual recidivism, although few have questioned how adequate the measure of sexual recidivism is and whether it can be used for all offenders across all jurisdictions (Langevin et al., 2004; Lussier & Cale, 2013). That said, researchers are now generally well aware that there is no true base rate for sexual recidivism—there is no single rate or percentage that can be applied to all individuals convicted of sex crimes. Detailed examinations of recidivism rates have clearly shown that the base rate of recidivism can vary drastically with changes in parameters such as the measurement criteria selected, the length of the follow-up period, the nature of the sample, etc. As Rice and Harris (2006) point out, the base rate of recidivism is a function of the question raised and requires risk assessors, analysts, and researchers to be specific about who is being considered and over what follow-up period. In the 808 empirical studies reviewed,^2^ the reported percentage for general recidivism (i.e., any criminal reoffense) varied between 0 and 90%, while the reported percentage for sexual recidivism varied between 0 and 68%. The risk of recidivism is not absolute but context dependent and that context extends well beyond offender characteristics.

### Recidivism Rates Vary According to the Length of the Follow-up Period

**Figure 4.**
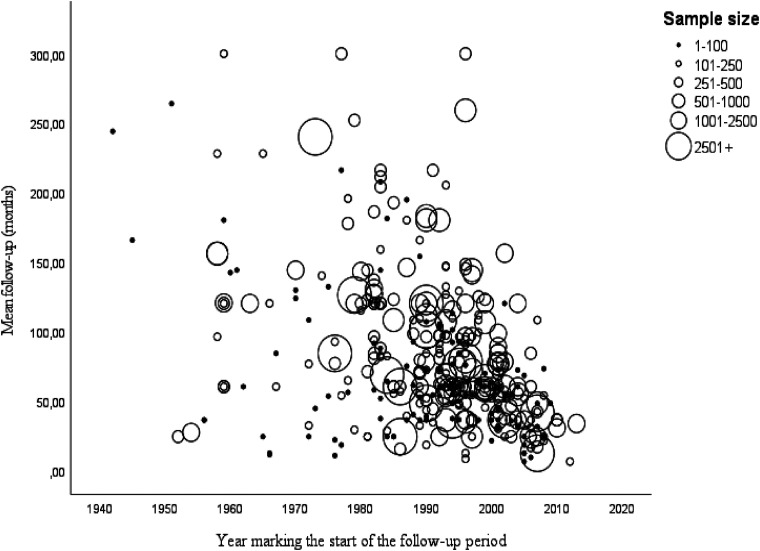
Average length of the follow-up period in recidivism studies.

*Notes.* The size of the bubble reflects the sample size used in recidivism rate estimates. This chart covers all the empirical studies during the 1950–2019 period that met our criteria. The kappa coefficient for the measure of the follow-up period was .710 (SE = .015).

There is a consensus among researchers that recidivism rates for a given cohort are a function of the length of the follow-up period considered: the longer the follow-up period, the higher the recidivism rates. The follow-up period is used by researchers to establish the window of opportunity for a criminal offense. By convention, this period starts when an offender is back in the community—when the sentence ends, when the offender is paroled, or at the start of a community-based sentence (e.g., probation, fine, conditional sentence)—and ends when researchers begin to collect information on recidivism. Not only can the follow-up period vary across cohorts, it can also vary across offenders within a given cohort. Failing to take this into account can lead to underestimation of recidivism rates (e.g., Soothill & Gibbens, 1978). Our examination of the scientific literature found that the follow-up period in studies varies from a few months to about 25 years (Figure 4), with the majority of empirical studies based on an average follow-up period of about 8 years. Studies with a longer follow-up are not only limited in number but are generally based on very small samples.

### Recidivism Rates Are Associated to Some Individual-Level Characteristics

The idea of a single base rate for sexual recidivism was finally put to rest by the discovery that recidivism rates vary according to various offender characteristics (e.g., Hanson & Bussière, 1998; Proulx et al., 1997; Quinsey et al., 1995). Identification of those individual characteristics has received a great deal of attention from researchers, who tend to refer to them as predictors or static/dynamic risk factors. These risk factors have been found to be statistically associated to rates of recidivism in studies using a prospective research design and a number of meta-analyses have also helped establish their empirical status (e.g., Hanson & Bussière, 1998; Hanson & Morton-Bourgon, 2005; McCann & Lussier, 2008). Risk factors for SOR include an individual's criminal history (e.g., prior conviction for any crime), past sexual offending (e.g., prior conviction for a sex crime), victim characteristics (e.g., male, less than 12 years old), mental health (e.g., personality disorder, psychopathy), and psychological, sexual, and social functioning (e.g., cognitive distortions supportive of rape and sexual assault; e.g., Craig et al., 2005). These factors are usually the foundation for the development of risk assessment methods and tools. While some of the most robust factors predictive of SOR are the same as those found in general samples of offenders (samples that are not limited to offenders who have committed sexual offenses; e.g., Gendreau et al., 1996), a subset of factors associated more specifically with sexual recidivism has been reported. For example, it has been shown that some of the best predictors of sexual recidivism include measures of deviant sexual preferences based on phallometric data (e.g., Hanson & Bussière, 1998). More specifically, while not all convicted offenders present deviant sexual preferences, those who do are more likely to have been arrested/convicted for a sex crime. This is especially true for offenders involved in sexual offenses against children. However, the risk factors empirically associated with recidivism provide the most fundamental basis for examining differential recidivism risk probabilities between offenders (e.g., Hanson & Morton-Bourgon, 2005). These differential probabilities are generally expressed and examined either along a continuum of risk probabilities (Quinsey et al., 1998) or in terms of various risk bins or risk categories (e.g., very low, low, moderate, high).

### Recidivism Rates Are Statistically Associated With Criminal History Indicators

In the field of prediction, past behavior is considered one of the best predictors of future behavior (Nagin & Paternoster, 2000), and this observation also holds to a certain degree for individuals convicted of sex crimes and for sexual recidivism. A number of features in an offender's criminal history provide information about the probability of sexual recidivism. For instance, the number of previous convictions for sexual offenses and the characteristics of those offenses (e.g., sex crime against a male victim, use of violence) are statistically predictive of sexual recidivism (Hanson & Bussière, 1998). Surprisingly, this statistical association has not been the subject of much theorizing and could be subjected to various interpretations (e.g., however, Brouillette-Alarie et al., 2018). While aspects of criminal history are considered static predictors of sexual recidivism because technically they do not change once present, their statistical association is subject to change over time. For example, older convictions are less predictive of recidivism than more recent ones (Amirault & Lussier, 2011). Criminal history indicators tend to be more effective in predicting risk than clinical indicators for several reasons. First, relevant clinical indicators (e.g., deviant sexual preferences, lack of self-control and impulsivity, cognitive distortions supportive of sexual offending) are more difficult to assess than criminal history indicators based on criminal justice data. Second, criminal history indicators are more directly related to sexual offending than clinical risk factors (i.e., crime predicts crime). However, criminal history indicators and sexual recidivism are subject to some of the same measurement errors (e.g., the likelihood of a crime being reported to the police, quality of police investigation). Clinical risk factors (e.g., low self-control) are less likely to share the same measurement errors with those of sexual recidivism (e.g., being convicted of a sex crime). As a result, the overlapping measurement errors can artificially inflate the statistical association of two indicators based on the same source of information.

### Sex Offender Recidivism Is Not Limited to Sexual Recidivism

Because it has been assumed that recidivism among individuals convicted of a sex crime reflects a specific predisposition for sexual offenses (see Guttmacher & Weinhofen, 1952), research on SOR has traditionally been focused on this group. This assumption has, however, been challenged on several grounds. When homologous recidivism (i.e., recidivism involving the same offense type) is examined, sexual recidivism rates are not just relatively low but some of the lowest across types of crime (e.g., Langan et al., 2003). Descriptive studies of samples composed of perpetrators of sexual offenses have shown that their criminal history is characterized mainly by nonsexual crimes (Lussier, 2005). Not only the prior record of recidivists is composed mainly of nonsexual offenses, their criminal recidivism is more likely to be for a nonsexual offense than for a sexual offense and this is especially true for adolescents (Caldwell, 2010). While the etiological factors responsible for this remain relatively unclear, studies that involve factor analysis have consistently identified an “antisocial” component underlying the risk factors for sexual recidivism (e.g., Allen & Pflugradt, 2014). Relatedly, others have argued that these findings reflect well-documented observations about nonsexual offenders that show that offending, when it persists over time, takes different forms (e.g., Lussier et al., 2005).

### Recidivism Rates for Adult Offenders Vary According to Age

Another key factor that can influence recidivism is age, more precisely age at release. The role and importance of age are well documented in criminology from theoretical, empirical, and methodological standpoints (e.g., Farrington, 1986) and suggest that offending is age dependent. For example, rates of crime tend to peak during adolescence and decrease after adulthood is reached. These general trends were overlooked in the early research on SOR, usually due to the assumption that the propensity to reoffend was relatively fixed and stable over a life course (see Pithers, 1990). Examination of recidivism rates according to offenders’ age at release generated much-needed debate and research that led to certain adjustments to risk assessment tools (see Lussier & Healey, 2009). Researchers noticed that, among other things, risk assessment tools were skewed by information about younger adult offenders whose probabilities for sexual recidivism were higher, meaning that these tools generated biased estimates for older adult offenders (e.g., Barbaree et al., 2003). Research has shown that not only is the age at release a statistical, albeit modest, predictor of sexual recidivism, but that the base rate of sexual recidivism should be age adjusted (e.g., Abbott, 2011; Wollert, 2006).

### The Specificity of Adolescent Offenders

Adolescents who have been sexually offended have mistakenly been viewed as inevitably becoming adult perpetrators of sexual offenses (e.g., Letourneau & Miner, 2005). Research findings, however, show that it is imperative that adolescent and adult sexual offending are considered as relatively distinct phenomena (e.g., Lussier & Blokland, 2014). The distinction goes well beyond the issue of the continuity of sexual offending over time (McCuish & Lussier, 2017). For example, researchers have stressed that there are differences between adolescent and adult offenders in terms of sexual recidivism rates (Caldwell, 2002). Although there is a good deal of variation between studies, a meta-analysis by McCann and Lussier (2008) shows that, over a comparable follow-up period, sexual recidivism rates tend to be lower for adolescents than those reported for adults, while rates of general recidivism tend to be higher. Concepts and knowledge from studies based on adult offenders cannot simply be imported into discussions about adolescents. In order to untangle the available information about adolescent and adult sexual offending, it is important to recontextualize recidivism within developmental contexts, considering maturity and psychosocial development (e.g., Caldwell, 2010; Smallbone, 2006). Researchers need to provide more information about these areas and contextualize sexual recidivism within developmental stages and their associated tasks and challenges (e.g., development of self-control, sexual development).

The widely accepted conclusions of SOR research regarding risk and sexual recidivism are in sharp contrast to some widely held beliefs about sex offenders, risk, and sexual recidivism (e.g., Lussier et al., 2021b). This is particularly the case for the conclusion that SOR has both static and dynamic aspects. These different aspects suggest that there is both continuity and discontinuity in offending across ages and development stages and that continuity of offending is not necessarily characterized by a series of sexual offenses but can take many different forms. Given this, an exclusive focus on the risk of SOR may be too restrictive from theoretical, clinical, empirical, and crime-prevention perspectives. Two key research questions that remain relatively unexplored are why some repeat offenders commit both sexual and nonsexual crimes and why, for some offenders, offending might be limited to a certain life stage. Recognizing the continuity/discontinuity of offending and the general/specificity of recidivism further highlights the importance of the measures used to evaluate the key parameters of recidivism, such as indicators of recidivism and the length of the follow-up period.

## Sex Offender Recidivism and Unresolved Issues

While the number of publications on SOR has remained relatively constant since the early 2010s, the number of empirical studies has declined. Researchers appear to have been slowly moving away from the study of sexual recidivism rates and instead focusing on policies aimed at reducing these rates rather than dealing with a number of unresolved empirical issues. The discussion that follows is not exhaustive but highlights some of the key issues that still remain to be addressed.

### Current Measures Underestimate Sexual Reoffending, but to What Extent?

Researchers have long acknowledged that recidivism rates based on criminal justice data underestimate actual rates of reoffending. The key issue is to what extent criminal justice data underrepresents this rate, making the key research question how to determine the level of misrepresentation (also known as the dark figure of crime). While many researchers feel that inability to deal with the dark figure of crime is the cornerstone of all difficulties in SOR research, no one has been able to bring much clarity to the issue, which has led to three contrasting positions: (a) unilaterally rejecting current estimates based on official data; (b) acknowledging the limitations of criminal justice data and accepting them as inevitable, (c) continuing to use the data but estimating actual rates using various techniques. This issue is raised throughout the literature on sexual recidivism but few researchers have attempted to address it. Abbott (2020) discusses three methods used in research on the dark figure of crime: (a) self-report data (e.g., polygraph/lie detector research, Ahlmeyer et al., 2000); (b) life-time sexual recidivism analysis (e.g., Doren, 2010; Langevin et al., 2004), and (c) statistical modeling (e.g., probabilistic simulation; Scurich & John, 2019). However, these avenues are also characterized by significant and seldom-discussed methodological problems. In a recent study, researchers have raised two important issues related to the estimation of the dark figure of recidivism (Kelley et al., 2022): (a) The distribution of the number of undetected victims is highly skewed across convicted offenders suggesting that a small proportion of recidivists have a significantly higher proportion of undetected victims; (2) The proportion of undetected victims tend to drop after an initial sanction. These findings offer some directions as this topic deserves more attention, particularly from a policy perspective given the importance of decisions based on estimates of SOR. As well, the lack of clear-cut and unambiguous information may mean that personal judgments, values, and beliefs are able to affect the debate (e.g., Hanson, 2000). In the end, exploring the dark figure of crime may come to be considered taboo, with researchers avoiding the issue altogether because it raises doubts about the current foundations of SOR research.

### Are Individual Rates Stable Throughout a Life Course?

Research on the statistical association between age and recidivism suggests that recidivism rates decline over a person's life. This research is, however, based on cross-sectional observations, that is, observations that, for example, the probabilities of recidivism are higher for a 20-year-old offender than for someone 60 years old. It does not make it possible to determine what the risk probabilities are for a particular 20-year-old when that person reaches 60. Given this, can we truly speak of an aging effect? The first scenario deals with differential risk probabilities while the second is more concerned with a probability estimate of long-term risk. There are also several underlying factors that need serious consideration. For instance, do statistical age-recidivism findings reflect the effect of an age, a cohort, or even a period? An age effect means that as a person grows older the risk of recidivism changes because of factors related to aging, such as changes in opportunities to perpetrate a sex crime, influence of peers, or sexual drive, which may affect the risk of recidivism and the base rate of sexual recidivism for various age-groups (e.g., Barbaree et al., 2003). A cohort effect implies that those born at a particular point in time (e.g., in the 1960s) may behave differently, irrespective of aging, because of generational factors (e.g., gender roles, norms and beliefs, sexual education). Period-specific factors (e.g., a change in policy, access to technology and potential victims) can also impact sexual recidivism rates in ways that affect recidivism irrespective of age and cohort. Whether individual recidivism rates are reflective of an aging effect unaffected by cohort and period effects remains open to debate.

### Sexual Recidivism Rates Across Periods

Crime rates evolve over time due, among other things, to cohort and period effects. In North America from the 1970s until the early 2000s, there were significant changes in crime rates due to a combination of factors (e.g., Blumstein & Wallman, 2000). Crime rates, including those for sexual offenses, were dropping (e.g., Kong et al., 2003), raising the possibility that recidivism rates might have changed as well. This might have been due in part to the effect of sex offender laws, some of which had been in effect for over 70 years, which included various measures, programs, and policies aimed at preventing sexual offenses. Other significant societal changes (e.g., increased awareness of sexual victimization and its negative consequences) could have impacted recidivism rates, directly and indirectly. Not only is there increased societal awareness about issues involving gender roles and inequalities, sexual violence, and the experiences victims have with the criminal justice system but scientific knowledge about sexual offending, the perpetrators of such offenses, and the prevention of sexual recidivism has significantly improved. The presence of period effects remains relatively undocumented, although some research has found interesting trends (Lussier et al., 2022; Lussier et al., 2023a; 2023b). For example, for some time there have been reports suggesting that sexual recidivism rates are on the decline, leading to statistical adjustments to actuarial tables of risk assessment tools (see e.g., Donaldson & Wollert, 2008) and, more recently, Caldwell (2016) has suggested that sexual recidivism rates for adolescents have been declining over the past 3 decades (see, however, Lussier et al., 2023a). Such changes, if present and continuing, would have far-reaching implications. However, some have hypothesized that stringent sex offender laws may have increased awareness of the stigma associated with sexual offending to the point that victims may be less inclined to report victimization by family members or acquaintances to the police (Hlavka & Uggen, 2008). The effect could be even more pronounced if the offender is a minor.

### Accuracy of Long-Term Estimates of Sexual Recidivism

Generally speaking, short-term estimates of sexual recidivism are more useful in determining whether the risk associated with the release of an offender (e.g., parole) can be effectively managed in the community and there has been a bias in favor of short-term studies, given the time needed to conduct the longitudinal studies needed to establish long-term base rates. However, for several decades, experts have been asked to conduct risk assessment for the courts in cases where long-term legal dispositions are at stakes (e.g., sex offender registries, civil commitment, indeterminate prison sentence, imprisonment for public protection) (see Harrison, 2017). In that context, some researchers have raised the critical issue of how to estimate long-term base rates for sexual recidivism (e.g., Doren, 1998): given that the base rate of sexual recidivism is dependent on the length of the follow-up period, how high are those base rates after 15, 20, or even 25 years post-release? Recent studies using contemporary samples are inevitably based on short follow-up periods (Figure 4) and, as a result, are more at-risk for false negatives. Only a very limited number of studies have examined recidivism rates over long periods (Figure 4) and their findings often differ. For instance, some researchers have estimated that longer follow-up periods may show sexual recidivism rates that approximate or even surpass 50% (Harris & Rice, 2013), while others feel they will not come close to that level (Hargreaves & Francis, 2014). Long-term recidivism rates are more difficult to establish as not only is there an inherent bias in longitudinal studies related to the length of the follow-up period but offenders may abscond, be deported, or move out of state or out of the country. Researchers may also fail to account for periods when offenders have been prevented from offending by being hospitalized, incarcerated for long periods, or incapacitated for other reasons. The information needed for an accurate longitudinal study is often difficult to obtain, compile, update, and analyze over long periods. As a result, long-term projections can easily become unreliable. Furthermore, our examination of empirical studies shows that long-term estimates are not only based on much older studies, raising issues about cohort and period effects, but also on smaller sample sizes, raising questions about whether they can be generalized.

### Gender Differences in Sexual Recidivism Rates

Although there appear to be few gender differences in risk factors for SOR between men and women (Freeman & Sandler, 2008), there is a relatively clear trend showing that sexual recidivism rates for women, as a group, are lower than those reported for men (e.g., Cortoni et al., 2010; Sandler & Freeman, 2009). Differences in rates may be broad, reflecting gender differences and suggesting that women as a group are distinct from men in terms of their risk for sexual recidivism, or these observed differences may be a reflection not of between-group differences but of within-group differences. For example, there may be a small subgroup of men who have a higher risk of sexual recidivism while such a subgroup may be relatively absent among women. Group differences would then be an artifact of the rates for a small subgroup of men. Recidivism among female sex offenders has seldom been investigated and the studies that have been conducted have limitations such as small sample size which, when combined with the low recidivism rates observed, reduces the possibilities for statistical inference (e.g., Sandler & Freeman, 2009). Classification models have been proposed to better understand female sexual offending while accounting for observed heterogeneity (e.g., Sandler & Freeman, 2007; Vandiver & Kercher, 2004), but these models have usually been imported from studies on male sexual offending and, given the gender differences that have been observed (e.g., Marshall & Miller, 2019), it is reasonable to question their validity. It might be more appropriate to investigate female sexual offending by relying on concepts and knowledge from the literature on general female offending (e.g., Miller & Marshall, 2019; Sandler & Freeman, 2009). Current knowledge suggests that base rates of sexual recidivism estimated for men or derived from predominately male samples should not be extrapolated or generalized to women, suggesting that a different analytical strategy may be needed to study female sexual recidivism, one that considers its low occurrence (e.g., qualitative studies).

### Neighborhood, Regional, State, Country, and International Variations in Recidivism

**Figure 5.**
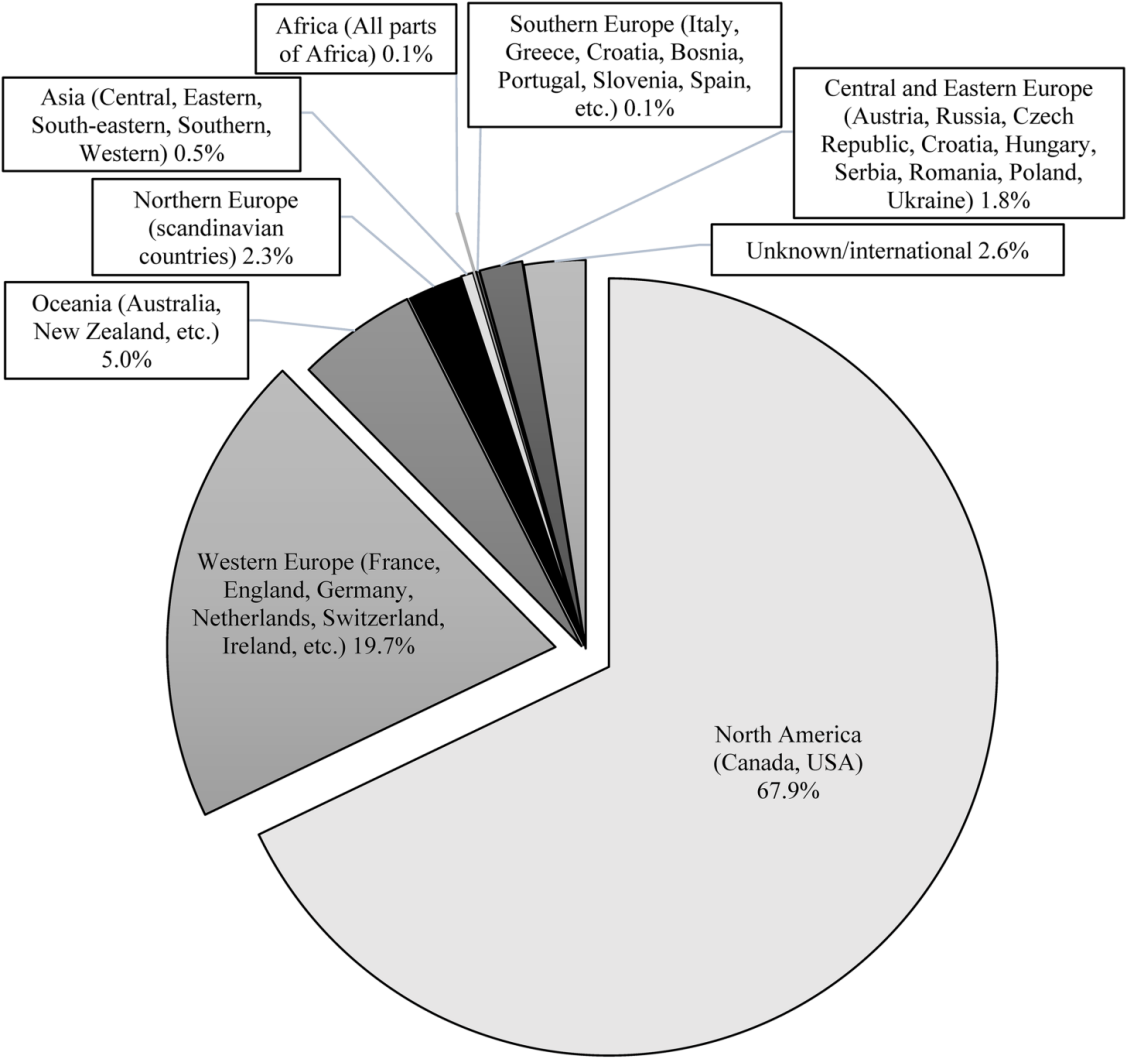
SOR research by region in which the study was conducted.

*Notes*. Based on empirical studies only. The kappa coefficient for this variable was .865 (SE = .035).

Two thirds of the empirical studies in our sample were conducted in either the United States or Canada (Figure 5). Prior to the 2000s (based on the publication year), SOR research was even more predominantly American and Canadian (about 77% of all SOR publications). This trend seems to have changed in the past 20 years, probably in part because of the specificity of the American criminal justice system and its specific response to prevent sexual reoffense. The specificity of American laws dealing with justice-involved perpetrators of sexual offenses (e.g., public notification, public sex offender registries) seriously limits the possibility of generalizing the results of SOR research beyond the state where the study was conducted. This specificity has unintentionally raised awareness of geographical issues related to SOR in two distinct ways: (a) the expansion of the non-US-based SOR research; (b) the examination for specific locations where offenders live and perpetrate their sexual offenses. Indeed, the relative proportion of non-US studies has significantly grown since 2000. The relative proportion of publications from Australia and New Zealand has gone up from about 1% (1940 − 1999) to about 6% (2000 − 2019) of all SOR research. In comparison, studies from the United Kingdom have significantly grown in number but not in proportion (about 10%) over the past 20 years. Since 2010, SOR research has also significantly increased in European countries, especially the Netherlands and Germany. This growth is essential and critical for allowing international comparisons, a very limited area of research.

The specificity of American laws and, in particular, public sex offender registries have also directed attention to the specific locations where offenders live and the proximity of the residences of convicted offenders to certain zones and areas (e.g., public parks, schools and daycares, bus stops) has sparked public outrage and favored the enactment of residential restriction laws (Zgoba, 2017). While these laws are not supported by empirical research on the environmental aspects of sexual offending (e.g., Calkins et al., 2015), they have led researchers to examine, among other things, community response to the re-entry of convicted offenders following their release. American researchers in particular have documented instances where offenders and their family members were subjected to harassment, bullying, eviction, and other forms of social stigmatization. It has been argued that the combined effect of public registries and residential restriction laws has been to force offenders and their family members to move into disorganized and criminogenic neighborhoods (e.g., Mustaine et al., 2006). These issues highlight the growing importance of examining neighbordhood and regional variations and disparities in sexual recidivism rates. For example, is it possible that these laws have crime displacement effects, with sexual offenses not declining but simply being committed elsewhere? Are these offenses being committed in areas where formal and informal sources of social controls are inadequate and citizens are more vulnerable and less likely to report their victimization? There are also relatively undocumented issues around possible disparities between urban and rural areas in terms of community re-entry experiences as well as the social services available to individuals who often have multi-needs (e.g., alcohol/drug abuse, anger management issues, mental health problems, intellectual disabilities). Nearly a century of work by environmental criminologists has shown that even in the city there are important variations in neighborhoods that can influence crime rates (e.g., Brantingham & Brantingham, 1981) and create hot spots for crime (e.g., Sherman et al., 1989).

Beyond the more widely accepted conclusions about SOR, a number of issues remain unresolved due mainly to a lack of research, contradictory findings, or simply a lack of data. These unresolved issues highlight the complexity of the phenomenon and the importance of contextualizing it across groups, time, and space, pointing out some of the challenges that confront researchers. Resolving these issues is complicated by the interplay of methodological issues, challenges, and limitations.

### Methodological Issues, Challenges, and Limitations

Researchers studying sexual recidivism face a number of methodological issues and constraints (Furby et al., 1989; Webster et al., 2006), some of which are related to the measurement of crime in general (e.g., Mosher et al., 2010), while others are more specific to the methodology and study design involved in conducting empirical research on criminal and sexual recidivism. Lösel and Schmuker's (2005) meta-analysis of the impact of treatment on sexual offense recidivism highlights the importance of methodological issues, showing that most of the “sex offender treatment effect” can be attributed to study, not treatment, characteristics. Methodological issues underlying the long history of research on criminal recidivism have been well documented. For example, given that researchers often rely on criminal justice data to measure recidivism, criminologists have reasonably argued that their results then reflect, at least in part, both the criminal justice system's performance in response to crime (e.g., Maltz, 1984) and the level of social organization, collective efficacy, and regulation of crime by formal and informal sources of control (e.g., Browning et al., 2004). This includes, among other things, whether a crime is reported to law enforcement, how this complaint is investigated by the police, identification of a suspect, the process and considerations involved in collecting evidence and laying criminal charges, the collaboration of the public in locating and finding the suspect, etc., raising methodological issues that, for various reasons, may not have received sufficient attention in research on SOR.

### Attrition Within the Criminal Justice System

Processing sex crime cases within the legal system involves a process of attrition, so only a certain number of complaints made to law enforcement agencies ends with a criminal conviction in a court of law (e.g., Daly & Bouhours, 2010). And much has been written about the police culture of disbelief (e.g., attitudes toward victims) and how it can affect the handling and investigation of complaints about different types of sexual offenses (e.g., see Thomas, 2017). Police agencies have seen their role in dealing with sexual offenses increase since the 1990s, especially in the United States, with the enactment of sex offender laws (e.g., criminal background checks, registration). Yet little is known about how law enforcement agencies use these tools and how they are perceived by police officers and investigators (Harris et al., 2018). For instance, sex offender laws that require frequent contact between registered offenders and law enforcement may lead some offenders to see police officers as friends or confidants (Powell et al., 2014). The way the law is applied may also lead to changes in the data. For instance, plea bargaining, in which the charge is changed to a less serious but related offense in exchange for a guilty plea by the accused may mean that data on recidivism based on convictions is less reliable than data based on arrests. Another factor involves statutes of limitations (e.g., Connolly et al., 2017). While not all jurisdictions have such statutes for sex crimes (e.g., Canada), those that do stipulate a legally defined window during which a perpetrator can be charged for a crime. This window (e.g., maximum 10 or 21 years), its application, and how it is calculated vary across jurisdictions, further complicating the evaluation of its importance on measurements of sexual recidivism. The statute of limitations can become a significant factor under certain circumstances—for example, cases where the offense occurred when the victim was a child but the victimization is not disclosed until much later (e.g., Mathesius & Lussier, 2014). Charges may also be delayed because of a backlog in DNA forensic testing (e.g., Wang & Wein, 2018). A statute of limitations could lead to further underestimation of sexual recidivism rates for those who manage to escape detection for long periods. While researchers are aware of these issues, their impact on recidivism rates remains relatively unknown, largely because of the difficulty in estimating their effect, which would essentially require researchers to measure the effectiveness of the criminal justice system and its handling of sexual offense complaints.

### Wide Range of Methodologies in Studies

SOR research is generally based on four methodological pillars: (a) a targeted population, (b) a longitudinal research design that includes a follow-up period, (c) a measure of recidivism and the source(s) of information used to measure it, and (d) an analytical strategy used to analyze the data. Behind terms such as “sex offender” and “sexual recidivism” are a wide range of realities and nuances that are lost in the operationalization process. There is also a great deal of variation in the methodology involved in the configuration and operationalization of the four pillars, as shown in Table 1, which highlights the various operationalizations of the concept of recidivism in our sample. The vast majority of studies rely on criminal justice data, with 45% of the studies basing the determination of recidivism during the follow-up period on a new arrest and 54% making this determination based on a new conviction. A very limited number of studies relied on indicators such as a new incarceration or a new police contact. Fewer than 5% of the studies relied on self-report data concerning a new crime. This raises further questions about both the search for a base rate of recidivism and the relatively quick and unexamined acceptance of conclusions about risk, risk factors, and risk management for this population. Researchers are faced with the absence of explicit guidelines about how to conduct a general criminal recidivism study or a study specifically concerned with sexual recidivism. In fact, our literature search did not find any specific guidelines that could help researchers maximize methodological rigor in this type of research. Not all SOR studies are created equal but this fundamental finding is rarely raised in the scientific literature.

**Table 1.** Sources of Information on Recidivism.

Measures and sources	*n*	%
*Criminal Justice System (courts, police, corrections)*		
Criminal justice professionals’ files (e.g., probation officers)	4	0.5
Police contact	19	2.4
Arrest/charge	362	44.8
Conviction	434	53.7
Incarceration	72	8.9
Unclear/unknown	15	1.9
*Other sources*		
Self-reports	30	3.7
Clinical files	3	0.4
Child protection services	7	0.9
Hospital files	1	0.1
Report from other institutions (e.g., military)	2	0.2
*Unclear/unknown source*	71	8.8

*Notes. N* = 808. These numbers refer to any measure of criminal recidivism (general, sexual, violent, sexual/violent, technical violations, etc.). They exceed the total number of studies as multiple indicators can be used in a single study. The mean kappa coefficient for the measure of recidivism is .677 (*SE* = .090)

### Missing Information

**Figure 6.**
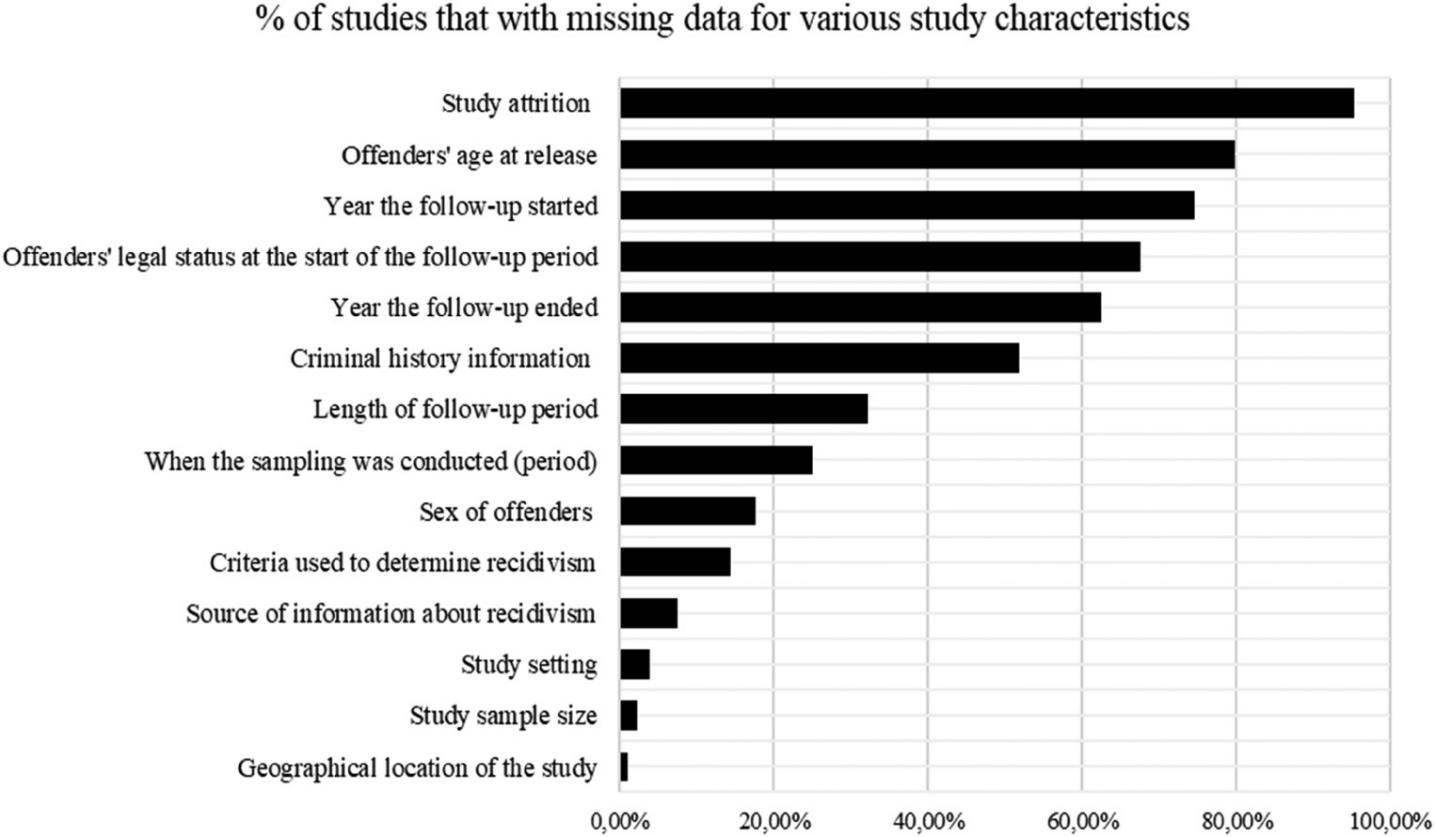
Missing data on key information in SOR studies.

*Notes.* The numbers (%) are based on empirical studies only. The mean kappa coefficient for these indicators was .702 (*SE*  =  .050).

In the context of this review, missing information refers to key pieces of information—such as methodological features of study design and recidivism measurement—that are either not clearly reported or not reported at all, limiting the possibility of replicating a study or generalizing study findings, two fundamental principles of the scientific method. Figure 6 highlights the prevalence of missing information in the 808 empirical studies we analyzed, showing that, for example, sample attrition, offender age at release, and the date of the first year of the follow-up period are rarely mentioned or discussed. It is vital that researchers properly contextualize their research, especially given that this type of research cannot be conducted under experimental conditions. What might appear to be contradictory findings between studies may simply reflect different methodological decisions or methodological limitations. Missing information about crucial elements may limit understanding of the context in which studies were conducted, making it difficult to interpret recidivism rates and their variations from one study to another.

### Reductionist Approach to Sexual Offending

The shift toward risk management of dangerous offenders has had an underappreciated impact on the study of sexual offending, leading researchers to focus on risk probabilities rather than the acts involved in sexual offending. Sexual offending encompasses a wide range of behaviors that range from gross indecency to sexually motivated homicide and include diverse behaviors such as child pornography (production, usage), rape and sexual assault, and child sexual abuse and sexual exploitation that vary in terms of nature, frequency, context, intrusiveness, and level of violence and harm. Even a legally defined crime such as sexual assault can vary along various dimensions (e.g., level of violence, level of physical injuries, use of a weapon, presence of accomplices, length of time over which the offense occurred). This wide range is lost in the aggregate measure “sexual recidivism.” Legal definitions of criminal offense do not include much about the actual criminal behavior perpetrated by an offender, such as the nature of the behavior and its context, the individuals involved (age, sex, relationship), the number of abuse events and duration of the abuse, level of violence and injury inflicted on the victim, or the level of sexual intrusiveness (e.g., physical contact with the victim, type of physical contact). The specific nature of the risk is lost in the legal process. We have found only 20 studies that provide specific information (e.g., number of victims, victim's age, victim's gender, level of violence use) about the characteristics of the sexual offenses being considered beyond a legal description of the offense (e.g., Howard et al., 2014). This is hardly a description of the “risk.” Furthermore, recidivism is usually defined in terms of an underlying, often unidentified construct (e.g., sexual offending, sexual deviance, sexual violence). This issue is further complicated because the behaviors defined as sexual offenses change over time and across generations (e.g., homosexual acts, sex crimes perpetrated abroad). Sexual recidivism is used as an aggregate measure that does not provide information about the perpetrator's actions and motives, the context in which those actions occurred, or their purpose.

### Pseudorecidivism

Pseudorecidivism refers to a situation in which an offender is rearrested/reconvicted for a crime/s committed prior to the index offense (Singh, 2014). For example, after an individual is investigated and convicted for a sexual offense, police may then discover other victims, leading to additional charges and/or convictions that take place after the index offense. Archival criminal justice data, which provides little contextualization for convictions, could suggest that these arrests or convictions were reoffenses. The level of pseudorecidivism remains largely unexplored, although it raises fundamental questions about the concept of sexual recidivism and highlights the gap between how this concept is used by the courts, those making risk assessments, and those studying criminal behavior. After all, in this example, the offender could legitimately be considered a recidivist, although not in the sense that the court and actuarialists applying this term understand it.

### Recidivism as a Limited Approach to Criminal Careers

Recidivism occurs at a specific moment in an individual's life. In the jargon, it is often said that the recidivism event is one “frame” within a film of an offender's life course. One critical issue is that criminal behavior at the time of arrest is not necessarily representative of an offender's criminal behavior over a life course. For example, when cohorts of offenders are considered based on the same criminal career event—first arrest for any crime—general criminal recidivism rates tend to increase as the number of arrests increases and then stabilize (e.g., Piquero et al., 2007). These observations show that there is both continuity (chronic offending) and discontinuity (desistance) in offending. Research in this area has been done mainly on general recidivism and rarely with samples of convicted sex offenders (see, however, Stander et al., 1989). Studies on sexual recidivism tend to pool offenders at different stages of their criminal careers: first-time offender, first-time recidivist, second-time recidivists, and so on (see, Duwe, 2012). In other words, they tend to look at aggregate recidivism rates, irrespective of offenders’ individual criminal careers (Lussier & Cale, 2013). Actuarial risk assessment instruments thus consider the criminal history of prior sex crimes to be a “static” risk factor for sexual recidivism rates (e.g., Hanson & Thornton, 2000), failing to consider the dynamic nature of offending.

### Post-Release Experiences

Recidivism studies examine whether a convicted offender has committed another sexual offense during a given follow-up period. The reality is, however, that we do not know a great deal about that follow-up period. How it is defined and measured raises numerous challenges that can impact the measurement of not only time-at-risk but also recidivism. The seminal work by Zamble and Quinsey (1997) details some of those challenges with respect to community re-entry and community reintegration (see also Laws, 1989). Later researchers have examined the follow-up period with two main goals in mind: (a) identifying the dynamic risk factors that can facilitate or precipitate a reoffense, (b) identifying the contextual factors that characterize the community re-entry of convicted sex offenders (e.g., eviction, harassment, loneliness), especially in the context of sex offender laws (e.g., Lasher & McGrath, 2012). Time-at-risk is the period during which individuals are actually at risk of perpetrating a sexual offense, a measure inherently part of the measurement of sexual recidivism rates. At a minimum, researchers should consider life events that can impact time-at-risk (e.g., periods of incarceration, deportation, emigration, moving out of a jurisdiction, death). Formal surveillance can limit a convicted offender's location and may also have an effect on recidivism rates through, for example, conditions imposed by a parole board, constant follow-ups with a probation officer, electronic monitoring, or intensive surveillance. Those situations are rarely considered in recidivism studies, leading to the possibility that the absence of recidivism will be interpreted as indicating that individuals are at low risk for offending rather than that risk is being effectively managed in the community. As the number of sex offender laws has increased over the years, estimating the time-at-risk has become increasingly challenging. Other measures may also limit offenders’ locations and impact the rates observed (e.g., residency restriction laws).

## Research Initiatives and Innovations

Social science research involves a series of compromises that are reflected in researchers’ decisions at every step of an empirical study. With the advent of the risk paradigm in the 1990s, researchers proposed what were then considered to be innovative studies. However, the issues, challenges, and limitations that were not addressed then have been carried forward by the next generation of researchers, raising serious doubts about the conclusions that can be drawn from the resulting work. While researchers are aware of these issues and limitations, few have critically and empirically examined the extent to which they affect the measurement of sex offender recidivism. There are, however, some notable exceptions, and a number of innovative research methods have been proposed.^3^ Over the years, researchers from different countries and jurisdictions have faced similar conceptual and methodological issues regarding the availability of data, so blaming them for all the limitations in the study of SOR would be unfair and unjustified. Institutions granting access to data for research purposes often limit the information made available, forcing researchers to address only certain questions or use a particular method (e.g., Morrow & Peterson, 1966). Researchers are frequently required to work with limited resources and information to which only minor additions and modifications can be made. However, if we look beyond the external constraints, researchers have been partially responsible for perpetuating the cycle of measurement error. Contemporary researchers are attempting to break this cycle by introducing research innovations and providing a new perspective on SOR. As Furby et al. (1989) note, at one point, measuring recidivism was itself a research innovation. The review that follows is not meant to be exhaustive and therefore excludes those innovations that are more practical and only indirectly related to the measurement of recidivism (e.g., risk assessment methods, risk prediction, communication of risk). Our focus is on research initiatives that are more clearly limited to the issue of measurement of sexual recidivism, some of which have received more attention (e.g., life tables) than others (e.g., capture–recapture; benchmark measures).

### Life Tables and Survival Analysis

Researchers measuring recidivism were quickly confronted with the issue of how to determine and work with different follow-up periods across offenders (e.g., 3 months, 2 years, 5 years). More specifically, researchers had to deal with the issue of false negatives (offenders who were incorrectly identified as non-recidivists). To address this issue, they turned to what is called survival analyses, life tables, or event history analysis (e.g., Cox, 1959), a set of methods initially used in the medical sciences that help adjust recidivism rates by considering differential follow-up periods and time-at-risk for individuals (e.g., Morrow & Peterson, 1966; Soothill & Gibbens, 1978). Survival analysis allowed researchers to account for the passage of time and examine how long an offender could “survive” a new arrest/conviction without committing another offense. This line of research was later expanded to examine the covariates of such survival (e.g., Hanson et al., 1995) and paved the way for the development of actuarial risk assessment. Life tables were initially used to adjust estimates of recidivism rates based on actual follow-up periods but also helped identify time-dependent survival patterns. More specifically, they showed that offenders were most at risk of recidivism in the period immediately following prison release. More recent research, however, has relied on life tables and survival analyses to address more complex questions about risk and whether risk is stable over time (Amirault & Lussier, 2011). For example, recent research found that the annual hazard sexual recidivism rate was the highest in the first year after their return in the community and that rate gradually dropped thereafter irrespective of offenders’ individual risk level, suggesting that the risk of sexual recidivism drops over time (Hanson et al., 2018). This type of research highlights the possibility of correcting offenders’ individual risk level (e.g., low, medium, high) by accounting for the number of years offenders have remained free from sexually offending.

### Capture–Recapture Methodology

One of the most elusive measurement issues in dealing with SOR is determining the risk of detection and apprehension. Although this accounts for some of the most critical limitations of sexual recidivism rates, it has not been the subject of much discussion or research. Some researchers have attempted to triangulate official information on sexual offending (e.g., new arrests for a sex crime) with other sources of information (e.g., self-reports), but these initiatives have been marginal and had very modest success. For example, Lussier et al. (2011) showed that if sources of information such as police reports or victimization statements, rather than the official criminal history indicators (e.g., past convictions), are considered, risk assessment tools such as Static-99 tend to show that those with the most arrests for sex crimes are at low or very low risk of sexual recidivism. They argue that relying on criminal justice data and indicators (e.g., past conviction) creates a bias in favor of offenders who have escaped detection until their initial conviction for a sex crime. Bouchard and Lussier (2015) propose an alternative approach to address the issue of risk detection, using capture–recapture methodologies to determine the risk of arrest and conviction. Widely used in biological sciences (e.g., Chao, 1987), the capture–recapture method allows social science researchers to estimate the size of difficult-to-reach populations and, in so doing, the risk of detection. Using that approach with criminal justice data and a sample of convicted offenders, they estimate that the risk of conviction for a sex crime is about 5%. To our knowledge, however, their findings have not been replicated. This sort of statistical modeling is flexible enough to be used with various samples and different types of data. While Bouchard and Lussier examined the general risk of conviction, they did not examine whether some offenders are better than others at avoiding conviction.

### Trajectory Analyses of Offending

Traditionally, research designs have focused on examining data related to only two time points: a baseline (e.g., prison release) and the end of the follow-up period. Researchers have then looked at the proportion of offenders arrested or convicted in the time between these two points. This research design fails to consider an individual's entire criminal career. Researchers have addressed this limitation by looking at life-course patterns of arrests and convictions for sex crimes using semiparametric group-based modeling (e.g., Cale et al., 2016; Francis et al., 2014; Lussier & Davies, 2011; McCuish et al., 2016). While the method has its own limitations, it provides a novel approach to an old issue. Examining the longitudinal sequence of arrest and convictions for sex crimes allows researchers to investigate whether there are trajectories of sexual offending (Tewksbury et al., 2012) and provides additional clarity about a number of issues, such as the number of patterns of offending, the prevalence of these patterns, the shape of offending trajectories, and patterns of desistance from sexual offending. Study findings suggest that (a) there is a relatively limited number of patterns of arrest/conviction for sexual offenses; (b) the pattern of arrests and convictions is age dependent in a number of ways, and (c) the pattern of self-reported sexual offending in community-based samples contrasts with the patterns seen for convicted offenders. Most importantly, this line of research highlights the importance of disaggregating information about offending and recognizing the role of age and the passage of time.

### Qualitative Examination of Desistance From Crime

The study of desistance among individuals convicted of a sex crime inaugurated research that has led to reconsideration of the almost exclusive focus on recidivism as a dichotomous phenomenon, requiring researchers to consider SOR and criminal recidivism more broadly. Recidivism is no longer seen strictly as a failure to avoid reoffending, but instead as part of a process characterized by lapses and relapses that culminate in the end of offending. An aggregate measure of criminal recidivism cannot, of course, detect the dynamic aspects of offending over time (e.g., deceleration, de-escalation, specialization). While the study of sexual recidivism had been highly focused on quantitative research methods, the study of desistance pointed to the need and importance of a phenomenological perspective that considers and values offenders’ perspectives (e.g., Harris, 2014). Considering the subject from an interest in desistance required breaking with traditional approaches and instead attempting to understand the process of change, including documenting offenders’ perspectives on the life events, life transitions, and local circumstances that can lead to changes in a given individual (e.g., self-identity), a decrease in rates of offending as the trajectory moves toward termination, and modification of what is required to maintain a non-offending state (e.g., Maruna, 2004; McAlinden et al., 2017). This is not to say that desistance research can only be done from a qualitative perspective but rather that qualitative studies provide a different and complementary look at these individuals, their reality and inner world, and their perception of their environment.

### Benchmark Comparison Measures

The criminal justice system has pressured risk assessors to provide assessments of the risk of sexual recidivism for particular individuals and in particular situations, leading to a number of new research questions. One underlying assumption about risk is that there is always some risk of sexual reoffending – the risk cannot be null given that offenders have committed at least one sexual offense in the past and past behaviors are the best predictor of future behaviors. While criminal justice policymakers sometimes suggest that ensuring the absence of risk is one of their goals when dealing with convicted offenders, such an objective is simply unrealistic given that the risk is not null for the general population (e.g., Malamuth, 1981; Marshall, 1997). As there is always some risk, baseline information and comparative figures are required to assist risk assessors in establishing the relative risk. A useful and novel approach was proposed by Nakamura and Blumstein (2016), who framed the research question as: how long does an individual convicted of a sex crime remain at a significantly higher risk for arrest for a sex crime compared to the general population in the same jurisdiction? Hargreaves and Francis (2014) estimate that the risk for sexually reoffending adolescent offenders converges with that of never-convicted youth after 17 years. They stress that the risk of sexual recidivism is not fixed and stable, as was once thought and, more importantly, they provide an analytical framework to quantity the process by which risk is determined. Using a similar approach, the study by Hanson et al. (2018) showed that within 10–15 years, the vast majority of convicted offenders with histories of sexual offending were no more likely to commit a sex crime than convicted offenders without such histories. The use of benchmark measures of risk based on data from comparison groups allows researchers clarifying the residual risk of recidivism for offenders with histories of sexual offending and move away from the simplistic depiction of risk based on myths and erroneous conclusions.

## Summary of Sex Offender Recidivism Research

The main objective of this study was to provide a critical and up-to-date review of the empirical scientific literature on SOR. This literature is uneven, at times fragmented, and characterized by a number of issues and challenges. Over the years, researchers have been repeatedly asked to provide a simple answer to a seemingly simple question: what are the recidivism rates for sexual offending? In response, the field has produced a wide range of findings that make it difficult to draw firm conclusions about SOR, leaving room for interpretation and personal biases (e.g., see Zapf & Dror, 2017). The wide variations in rates that have been reported for general and sexual recidivism are attributable to several factors. First, and foremost, researchers have clearly established that the risk of SOR is related to a number of individual factors, both static and dynamic. Prior studies have also shown that SOR rates vary according to factors that go well beyond those associated with the risk posed by offenders or their propensity to sexually offend. For example, studies based on samples of adult male offenders that use a longer follow-up period, consider arrests rather than convictions, and measure general rather than sexual recidivism tend to report higher recidivism rates. What remains to be clarified through research is the interplay between individual, contextual, and study characteristics. Researchers in the field have long recognized that the base rate of SOR is more effectively considered in terms of a series of questions and that, at a minimum, these questions should include the type of recidivism, with whom, over what period, and in what context. It is probable that risk assessors and researchers will soon be faced with contexts and situations that require a significant broadening of the concept of risk, something that the current research cannot address at this time. Some possible avenues for future research are considered in what follows.

There is a certain level of consensus about the importance and urgency of intervening to prevent a convicted offender from sexually reoffending. There is no consensus, however, as to how to accomplish this – what means to use, who should use them, and who should be the target of the intervention. Experts still do not agree on what constitutes a high risk of sexual recidivism or on who should be subject to the most stringent laws (e.g., Zgoba, 2017). Deciding these matters is critical given that recidivism rates are partly reflective of the functioning of the criminal justice system and its ability to accomplish a variety of objectives (e.g., public safety, retribution, deterrence, rehabilitation). Our study findings are more representative of research conducted in North America, especially the United States, and some of the conclusions may not generalize beyond those borders. However, even within those borders, there are important and significant variations from one jurisdiction to another, so the problem should be looked at in terms of state, region, city, and neighborhood. This point was not considered in the research we examined. As the number of international studies continues to grow (e.g., Cale et al., 2016; Maes et al., 2019; Rettenberger et al., 2015), careful attention to differences and variations can be expected to become a feature of this literature. Comparative international research on issues such as possible sociocultural differences in which sex crimes are reported to law enforcement agencies, criminal career patterns, variations in how criminal justice systems and police deal with sexual offenses, and measures taken to prevent recidivism should be on researchers’ agendas (e.g., Spaan et al., 2020; Lussier et al., 2023b).

In many ways, this field of research has become sufficiently mature that researchers can and should start investigating the complex issues underlying SOR, such as the effect of age, cohort, and period (Lussier et al., 2023b). The study designs chosen by researchers have made it almost impossible to disentangle these effects, raising concerns about whether findings can be generalized. For example, recidivism rates reported in the scientific literature 30 years ago, prior to the enactment of sex offender laws and the resulting increased number of arrests for sexual offenses, make little sense today. Long-term estimates of recidivism are generally based on older studies and often do not consider the context in which risk assessment and prediction were made. While there have been some attempts to determine long-term estimates of sexual recidivism, there has been little investigation of the effectiveness of these long-term estimates in forecasting future events. As study findings are based on retrospective data, estimates concerning future recidivism may have very little validity, given that they do not consider changes in the policies and practices of the criminal justice system over time.

Key innovations in this field of research have provided much-needed critical, complementary, and alternative viewpoints. Researchers, however, need to avoid being seduced by new analytical and statistical methods, particularly as some innovations fail to address fundamental problems in the measurement of recidivism (e.g., Lussier et al., 2019). For the field to grow, researchers need to tackle the issue of methodological rigor more directly. In our review of empirical studies, we noticed a lack of clarity about various methodological decisions that can lead to confusion and misinterpretation when SOR research is used by psychologists, psychiatrists, parole board members, lawyers, etc. While there are methodological rigor scales for a number of issues (e.g., Farrington et al., 2002), we know of no scale that can be used to assess the methodological rigor of SOR studies, assessing key features of methodology such as design, sample, sampling, measurement, and analyses. Such a scale would increase standardization in the field, enabling best practices that would also facilitate the interpretation and generalization of study findings. A similar initiative was proposed by a group of researchers who designed the RAGEE (Risk Assessment Guidelines for the Evaluation of Efficacy) statement checklist (Singh et al., 2014). This initiative highlights the importance of clarity and transparency by setting clear guidelines and standards for risk prediction studies and this includes the measurement of recidivism.

As diversity, equity, and inclusion are increasingly at the top of government agendas, research on SOR will be affected by these social movements. Canadian scholars have raised the critical issue that most recidivism research has involved the prevalent offender group—adult white men—leading to questions about the validity of results when applied to women and visible minorities (e.g., Hannah-Moffat, 2009). American scholars have suggested that the study of risk and recidivism could inadvertently contribute to maintaining social inequalities (e.g., Harcourt, 2015). Numerous questions could be raised about, for example, the generalization of recidivism rates for one segment of the population to other segments. There are also critical questions about whether sex crimes against visible minorities are less likely to be reported to the police. Parole board members, risk assessors, and other experts will have to address these emerging issues. Researchers need to be more proactive, thinking critically about how risk assessment may differentially impact offenders who are dealing with historical, structural, and social adversity and whether the science behind risk management strategies is potentially biased and damaging.

Another challenge for SOR research is the recent wave of social problems in the wake of the *#MeToo* movement. This movement not only signals that certain behaviors will no longer be overlooked or tolerated, it also highlights the presence of a subgroup of offenders that is seldom part of the existing body of research. The pattern of sexual recidivism in this group does not fit with the current portrayal of recidivism in several ways. For example, these offenders have managed to escape detection while sexually reoffending over long periods (Lussier et al., 2011), demonstrating the importance of a review of how recidivism is measured and the role and importance of delayed detection and differential risk avoidance, especially in a context where recidivism is generally studied using prospective follow-up periods and predicted using past criminal convictions/arrests. As a result, offenders who manage to escape detection in spite of a series of sexual offenses are likely to be seen by the criminal justice system as first-time offenders with a low risk of sexually reoffending (see Lussier et al., 2011). The key concepts in past studies and present actuarial risk instruments are incapable of addressing the risk posed by individuals who sexually reoffend over long periods without being detected or arrested.

Finally, a significant issue that limits the growth of this field of research is a lack of theorizing. Few researchers have attempted to develop a complete theory of SOR, focusing instead on the offending process that leads to sexual recidivism (e.g., Pithers, 1990; Proulx et al., 2014) or on the psychological factors involved in predicting risk (Beech & Ward, 2004; Mann et al., 2010). There have been some conceptual innovations stemming from empirical studies on sexual recidivism, but these are subject to the limitations of the measure on which they are based, raising the possibility that this research reflects the way the criminal justice system deals with some offenders rather than rates of recidivism for all offenders. The reluctance of researchers to explain how and why risk factors are predictive of sexual recidivism indirectly contributes to the myths and misperceptions about SOR. While theories are particularly needed for three interrelated aspects of sexual offending over a life course—persistence, escalation/de-escalation, and desistance (Lussier, 2017)—there has been little theorizing about these issues over the past 7 decades (see however, Smallbone & Cale, 2015). Sexual recidivism is currently investigated not as a theoretical or a clinical concept but to satisfy the demands of policymakers and correctional agencies for an affective mechanism to assess criminal justice practices. This field of research may soon find itself at a dead end without serious reconsideration of how to provide studies that are better tailored toward the explanation of the phenomenon, which may require dealing with more theoretically driven concepts (e.g., offending trajectory, risk avoidance, delayed detection, desistance). Increasing the number of conceptual innovations dealing with the issue of desistance is one example of an approach that could help the field develop on a solid and rigorous base.

## Conclusion

Research on SOR has been characterized by a natural evolution, imperfect and uneven, in a context where the criminal justice system has increasingly pressured professionals to establish the risk of recidivism. These pressures exceeded the ability of researchers to meet them, which might explain why fundamental flaws in the measurements central to this field have not been addressed. While the empirical contribution of actuarial studies is undeniable, one unintended consequence has been a slippage in the idea of risk. Risk refers to the nature of a threat, the probability that a threat will become reality, the factors that are informative about a threat, and the actual outcome resulting from a threat. The umbrella idea of risk for “sexual recidivism” unintentionally captures various realities that are lost in the process of analysis. For example, it does not deal with the qualitative aspect of risk, perhaps better conceptualized as the type of threat (e.g., type of abuse, modus operandi involved, target, risk of harm). With Western societies continuing to recognize a growing number of sexual behaviors as criminal, the term sexual recidivism will become even less clear. Against the backdrop of evolution marked by issues and challenges as well as innovation and progress, researchers are still faced with some of the issues and dilemmas that were being raised 7 decades ago. Reflecting on this situation, Keith Soothill (2010) suggests that this field of research has been built on shaky grounds. Sexual recidivism is a measure that forced its way onto researchers’ agendas due to pressure from the public, the media, victims’ rights movements, and various governmental agencies. Recidivism became combined with hypotheses about sex crime specialization, creating restrictive views of a phenomenon that remain difficult for experts to describe, measure, explain, and forecast. Scientific advancements in knowledge about risk assessment, protective factors, and communication of risk are tied to the notion of sexual recidivism and, as a result, suffer from its shortcomings. For the field to grow, researchers will have to stop deflecting or avoiding measurement issues and challenges and confront them directly. No consideration of area under the ROC curve or *p* values will help surmount the methodological shortcomings in measures of recidivism. The field has matured sufficiently that it is time to not only examine the usefulness of guidelines for conducting such research but to see that reports of this type of research are available in the scientific literature.

## Notes

1. These databases included Grey Matters: a practical tool for searching health-related gray literature; Open gray; Publications numériques du Québec (BAnQ); Réseau Santécom; Réseau informatisé des bibliothèques gouvernementales: RIBG; Institut de recherche et documentation en économie de la santé: IRDES; Banque de données en santé publique: BDSP; RESSAC; Érudit; WHO-IRIS; Collection mémoires et thèses électroniques; ProQuest Dissertations & Theses Global; Thèses Canada; Theses.fr; DART-Europe; WHO MiNDbank; CrimDoc (Criminology Library Grey Literature); Conference Papers Index; EThOS (Electronic Theses Online Service); GreyNet International; Networked Digital Library of Theses & Dissertations; Google Scholar,;and ResearchGate.

2. Conducting pooled estimates of recidivism to examine the average base rate across studies was not part of the present study but will be examined in a future study.

3. It was not possible to adequately cover all research initiatives and innovations. One issue that was omitted from this review is the use of Bayes Theorem and Bayesian inference (e.g., Elwood, 2018; Wollert, 2006) and its application in court cases involving sexually violent predators (Donaldson & Wollert, 2008), although the applications have been debated (e.g., Helmus & Thornton, 2016).

## Appendix I. List of keywords used to identify sex offender recidivism research

**Table:**

Keywords 1 “offender”	"sex* offender” OR “sex* aggressor” OR “sex* criminal” OR “child molester*” OR “rapist” OR “sex* assaulter” OR “sex* abuser” OR “sex* murderer” OR “child molester” OR “child molestation” OR “sex* offen?e” OR “perverted sex* behavio?r” OR “incest*” OR “sex with minor” OR “intercourse with minor” OR “sex* aggression” OR “sex* crime” OR “sex* abuse” OR “sex* exploitation” OR “sex* harass*” OR “sex* homicide” OR “sex* murder” OR “sex* batter*” OR “rape” OR “child* pornography” OR sexual abnormalit*” OR “sex* pervert” OR “sex-pervert” OR “sexual perver*” OR “dangerous offenders” OR “sex* deviant” OR “sex* devianc*” OR “sex* perversion” OR “sex* sadism” OR “sex* interest in child*” OR “courtship disorder” OR “paraphilia” OR “hebephilia” OR “teleiophilia” OR “exhibitionism” OR “voyeurism” OR “frotteurism*” OR “pedophilia” OR “sexual psychopath” OR “sexual criminal psychopath” OR “sex* predator” OR “ sexually violent predator” OR “hebephile” OR “teleiophile*” OR “exhibitionist” OR “voyeur” OR “frotteur” OR “paraphiliac” OR “sexual psychopath” OR “sexual criminal psychopath” OR OR “sex* assault” OR “sex* charge” OR “sex* delinquency” OR “indecent exposure” OR “gross indecency” OR “indecent assault” OR “carnal knowledge” OR “child* luring” OR “indecent behavior” OR “sexual felony” OR “unlawful intercourse” OR “unlawful sexual intercourse” OR “sex* delinquent” OR “sex* coercion” OR “sex* coercive” OR “sex* violence” OR “sexually violent” OR “sex* misconduct” OR “sexual harm” OR “inappropriate sex* behavio*” OR “atypical sexual behavio*”
Keywords 2 “recidivism”	“recidivism” OR “recidivist*” OR “recidivate” OR “recidivation” OR “rearrest*” OR “re-arrest*” OR “new arrest” OR “reconviction*” OR “re-conviction*” OR “new conviction” OR “reincarceration*” OR “re-incarceration*” OR “new incarceration” OR “parole violation* OR “new charge” or “new sex* charge” OR “new police contact” OR “lapse” OR “relapse*” OR “re-lapse*” OR “repeat offender*” OR “repeat offending” OR “repeat rape” OR “repeat sex* abuse” OR “repeat sex* offending” OR “discharged” OR “reoccurrence” OR “re-occurrence” OR “prognosis” OR “prognostic” OR “rehospitaliz*” OR “rehospitalis*” OR “re-hospitaliz*” OR “re-hospitalis*” OR “treatment outcome” OR “treatment impact” OR “treatment efficacy” OR “treatment effectiveness” OR “effectiveness of treatment” OR “efficacy of treatment” OR “impact of treatment” OR “community failure” OR “supervision failure” OR “parole revocation” OR “parole revoked” OR “parole discharged” OR “parole violation” OR “technical violation” OR “follow-up” OR “prison release” OR “parole suspension” OR “return to prison” OR “program evaluation” OR “program efficacy” OR “program effectiveness” OR “program outcome” OR “risk prediction” OR “risk assessment” OR “risk management” OR “desistance” OR “persistence” OR “offending trajector*” OR “continuity” OR “longitudinal study” OR “longitudinal data” OR “prisoner re-entry” OR “community re-entry” OR “community re-entry” OR “prisoner reentry” OR “reoffend*” OR “re-offend*” OR “re-offen?e*” OR “reoffen?e*” OR “criminal career”
